# The timing and nature of marine ecosystem recovery following the Permian-Triassic mass extinction

**DOI:** 10.1038/s44185-025-00117-2

**Published:** 2026-01-31

**Authors:** Annabel L. Nicholls, Paul B. Wignall, Haijun Song, Jack O. Shaw, Andrew P. Beckerman, Alexander M. Dunhill

**Affiliations:** 1 School of Earth and Environment, University of Leeds, Woodhouse Lane, Leeds, UK; 2 State Key Laboratory of Microbiology and Environmental Changes, School of Earth Sciences, China University of Geosciences, Wuhan, China; 3 Museum of Natural History, University of Colorado Boulder, Boulder, CO, USA; 4 School of Biosciences, Ecology and Evolutionary Biology, University of Sheffield, Alfred Denny Building, Western Bank, Sheffield, UK

**Keywords:** Ecology, Ecology, Ocean sciences

## Abstract

The Permian-Triassic mass extinction (PTME; c. 252 million years ago) was the most devastating extinction event of the Phanerozoic, resulting in up to 90% of marine animal species becoming extinct and profound ecological changes from Palaeozoic to Mesozoic faunas. The eruption of the Siberian Traps Large Igneous Province caused a cascade of environmental effects such as extreme warming, ocean anoxia and acidification which collapsed Permian ecosystems and delayed recovery in the Early Triassic. However, uncertainty remains regarding the temporal dynamics and nature of ecological recovery following the PTME. Models attribute a slow stepwise recovery within marine communities, from primary producers to top predators, reattaining pre-extinction levels of ecological complexity by the Middle Triassic. However, global empirical data indicates the rapid recovery of multiple trophic levels albeit in the form of top-heavy, unstable Early Triassic ecosystems. Further research promises exciting opportunities to apply community ecology models to ever improving databases of fossil ecosystems spanning multiple palaeolatitudes to test fundamental questions regarding the nature and timing of recovery and whether it really was “recovery” back to pre-extinction states; or “restructuring” to new baselines of ecosystem complexity more reflective of modern marine ecosystems.

## Introduction

The most catastrophic mass extinction event in Earth history occurred at the Permian-Triassic boundary, 252 million years ago (Ma), where Palaeozoic marine faunas were almost completely wiped out with estimated levels of marine animal species extinction reaching 81–94%^1–4^. The Permian-Triassic mass extinction (PTME) coincided with the emplacement of the Siberian Traps large igneous province (LIP)^5^ which triggered a complex cascade of climatic, environmental, and biological events on land and in the ocean^1,6^. In the marine realm, these processes are postulated to have driven extreme warming of ocean waters^7^, significant changes in nutrient input and productivity^8^, widespread ocean anoxia and euxinia^9^, and ocean acidification^10^.

There has been debate surrounding whether the marine extinction event occurred in two main pulses^11^, with the first pulse occurring during the latest Permian with great losses of species richness and the second some 60 ± 48 ka later in the earliest Triassic^12^ with further losses and community collapse^1,13^. Alternatively, others hypothesise that the extinction event played out as single pulse^14^ or longer “interval” of extinction lasting less than 200,000 years^15^. Extinction selectivity across the PTME has been explained by a combination of lethally warm shallow-ocean temperatures and widespread anoxic deeper waters^16^. Rates of extinction were generally very high across all latitudes^17,18^ albeit with evidence for slightly elevated rates of extinction at lower latitudes^19^ (particularly amongst pelagic organisms like ammonoids and conodonts) or higher latitudes^20–22^ (especially amongst benthic taxa like brachiopods and bivalves) albeit depending on differing methods (i.e. extinction vs extirpation) or time binning of data. Groups that completely disappeared across the PTME included the eurypterids, acanthodians, trilobites, rugose and tabulate corals, fusulinid foraminifers, and blastoid echinoderms^1^. Other groups suffered catastrophic losses, such as ammonoids^23^, brachiopods^24^, bryozoans, crinoids, and sponges^11^ whilst bivalves^25^, gastropods^11^, conodonts^26^, and fishes^27^ experienced moderate to severe extinction rates^28^.

Whilst the causes, magnitude, and apparent selectivity of the PTME has received an intense level of attention over the past decades, less has been afforded to the post-extinction interval and a greater deal of uncertainty remains around the timing and nature of marine ecosystem recovery^29^. Despite ongoing debate surrounding this uncertainty, it is widely accepted that the recovery from the PTME was unusually long compared to most other major Phanerozoic extinction events (see Erwin^30^ for a review of Phanerozoic mass extinction recovery rates)^31,32^. This slow, protracted recovery has been tentatively explained via competing, but not necessarily mutually exclusive hypotheses^29,33^. **(i)** The magnitude of the PTME and ecological disruption were so great that persistently low levels of alpha and beta diversity contributed to reduced biotic competition. This “ecosystem undersaturation” drove a suppression of diversification rates^29,34–36^. **(ii)** Prolongation of the environmental stressors (i.e. extreme heat, ocean anoxia, and ocean acidification) that caused the PTME continued throughout the Early Triassic^29,31,37–39^. **(iii)** Environmental instability and episodic occurrences of further strong environmental disturbances throughout the Early Triassic caused additional extinction events that suppressed, delayed, or even completely reset recovery^7,23,26,29,40^.

This perspectives piece aims to evaluate the current understanding of the nature and timing of marine ecosystem recovery following the Permian–Triassic mass extinction, and to highlight future research directions that could address ongoing knowledge gaps through innovative methodologies. In addition to answering why recovery from the PTME took so long, we also need to examine the progressive nature of ecosystem recovery following the extinction event and this depends heavily on how we define and measure recovery itself. Understanding the rebuilding of marine ecosystems after Earth’s greatest biotic crisis is key to anticipating how biodiversity and ecosystem function respond to major environmental perturbations^10^.

## What do we mean by ecosystem recovery and how do we measure it?

Recovery, in an ecological sense, is considered to be return to pre-disturbance levels of species diversity, structure and functioning within an ecosystem^41–43^. In the fossil record, this can be defined as the reappearance of highly diverse communities with a complex structure that are stable across macroevolutionary timescales^44^. Although seemingly straightforward in principle, assessing recovery from a mass extinction in terms of the re-attainment of taxonomic (i.e. generic or family level) diversity to pre-extinction levels directly from the fossil record has the considerable challenge of considering the effects of sampling and preservational biases. Defining when full ecological recovery has been achieved presents even more challenges on top of those presented by the limitations of the fossil record as there is no standard defined approach to quantifying ecosystem structure and function.

**Fig. 1:**
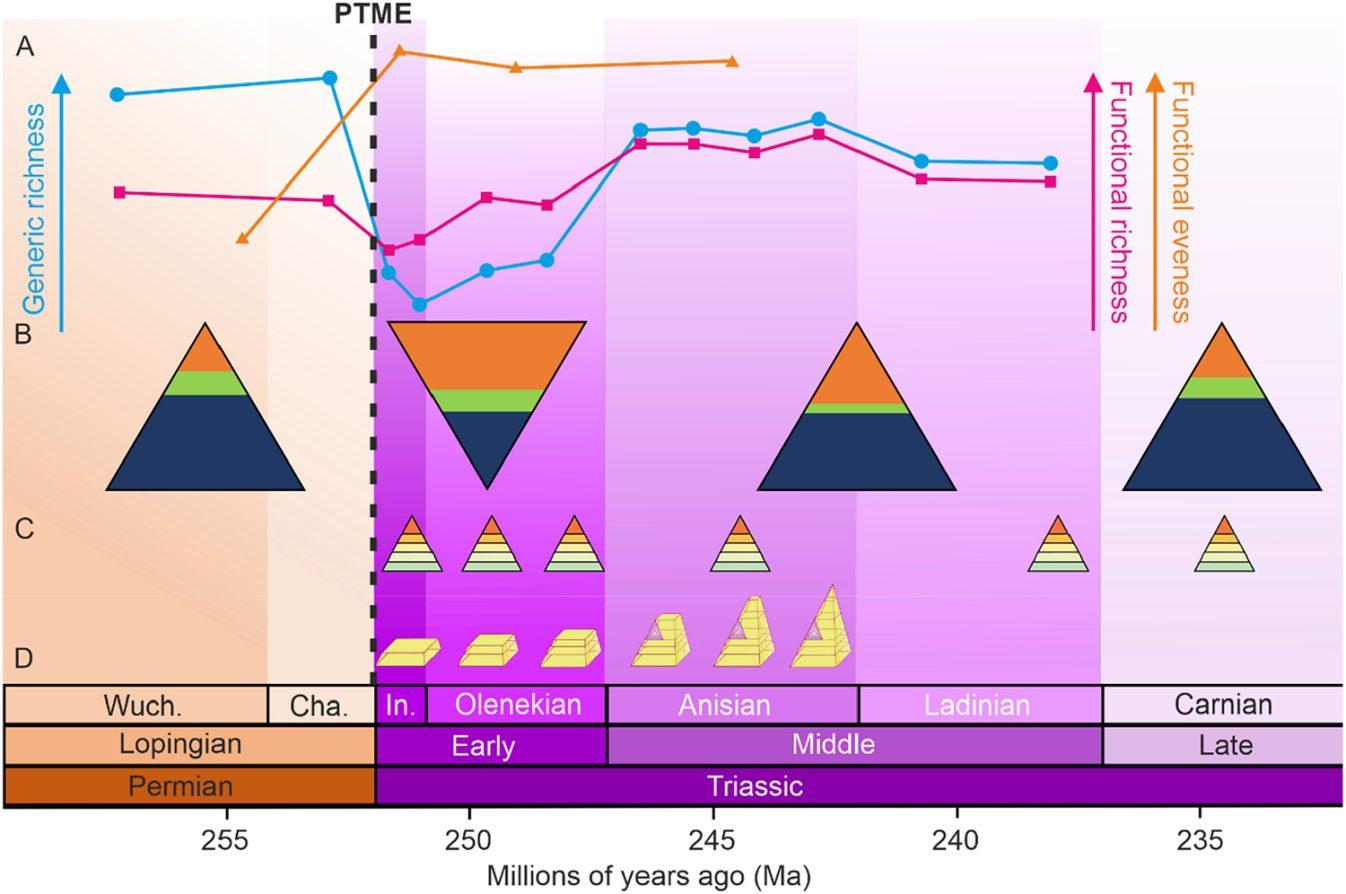
Schematic showing Lopingian and Triassic trophic pyramids and ecological metrics showing ecosystem recovery and competing hypotheses for ecosystem rebuilding in the aftermath of the PTME. **A** Generic richness (blue line)^47^ and functional richness (pink line)^47^ and functional evenness (orange line)^49^. **B** Functional pyramids showing the diversity of broad ecological guilds at the global scale at epoch level from Lopingian to Late Triassic^50^: Dark Blue = Non-motile benthic guilds; Green = motile benthic guilds; Orange = pelagic guilds. **C** Occurrences of lagerstatten that reflect seemingly complex communities of several trophic levels through the Early-Late Triassic^73^. **D** Conceptual model of stepwise trophic community rebuilding through the Early-Middle Triassic^69^.

Studies that have relied upon the re-attainment of pre-extinction levels of global species (or generic/familial) richness, identify that recovery from the PTME took at least 5 million years, with gamma diversity re-attaining latest Permian levels by the Middle Jurassic^45^ (if interpreting Sepkoski’s Compendium^46^ at face value), or sooner by the Middle Triassic^47,48^ (if using methods that correct for uneven sampling in the fossil record (Fig. 1A)). However, after major mass extinction events, the species assemblages of post-extinction ecosystems do not resemble that of pre-extinction ecosystems due to the high extinction magnitude and species turnover (i.e. up to 90% species extinction for the PTME) thus raising the possibility that the recovery of ecosystem structure and function did not follow the rebound of taxonomic diversity (i.e. species/generic richness). It has been postulated that taxonomic diversity could well have recovered significantly prior to the full recovery of stable and functionally complex ecosystems^49,50^.

Methods for quantifying functional diversity (i.e. the number and variety of ecological guilds) have been used in more recent studies of recovery from mass extinctions in an attempt to capture the timing and nature of ecosystem recovery^47,50^. In addition, others have used trace fossil diversity and abundance to track ecosystem recovery^39,51^ given that trace fossils represent an archive of (often soft-bodied) benthic activity and can be used as a proxy for both ecological diversity and abundance^51^. Global functional ecology studies suggest that only a very limited number of broad ecological modes of life were lost across the PTME^47^ (Fig. 1A), a pattern observed across other major extinction events (e.g. the Late Triassic^52^ and Cretaceous-Paleogene^53^). This observation has given rise to the *Skeleton Crew* hypothesis^47,52^ whereby high extinction rates drive species loss within each mode of life which drives a reduction in functional redundancy^54^. Consequently, global post-extinction assemblages are suggested to remain functionally rich but each mode of life is occupied by a small number of species (i.e. a “skeleton crew”)^47,52^. However, the same studies (i.e^47,52^) also identify differing patterns of taxonomic and functional diversity loss and recovery rates across different latitudes and ocean basins, hinting at differences in regional disturbances and recovery rates^19^. Ultimately, global analyses give estimates of global recovery but will struggle to capture the community-level variation in that signal and the trait-based methods commonly used to define ecological niches, or modes of life, are arguably too broad. However global ecological studies using this trait-based ecospace approach also suggest that ecological recovery can take much longer beyond the return to pre-extinction levels of taxonomic diversity^50^.

Whilst most studies have focused on ‘recovery’ of marine ecosystems to levels of complexity seen before the PTME, it has also been hypothesised that the aftermath of the PTME resulted in wholesale ‘restructuring’ of marine ecosystems^55,56^. This idea can be traced back to earlier attempts to characterise macroevolutionary patterns through the Phanerozoic with the switch from the Palaeozoic to Mesozoic faunas^45^ occurring across the Permian-Triassic (aka Palaeozoic-Mesozoic) boundary. Recent research supports this by showing that so-called ‘recovered’ marine communities in the Middle Triassic displayed much higher functional evenness than those of the latest Permian prior to the PTME^49^ (Fig. 1A). Ultimately, community structure is hard to measure in the fossil record as population sizes are hard to quantify, biotic interactions are uncertain amongst extinct organisms, and time averaging presents issues of uncertain community composition and thus the plausibility of faunal interactions. Some palaeobiologists have attempted to quantify changes in trophic structure across the PTME^13^, showing that taxonomic and ecological changes during the extinction phase were decoupled. This has not been tested during the longer recovery interval, however, the recovery of taxonomic richness and trophic structure have be shown to occur at different rates in the aftermath of the early Toarcian extinction event^57^, an event of much lower magnitude than the PTME that occurred in the Early Jurassic. In summary, evaluating and characterising change in biodiversity through time in fossil data requires integrating multiple metrics that describe the structure of the community, the number and identity of species and ultimately, where possible, relative abundances.

## A timeline of ecosystem recovery

In the immediate aftermath of the PTME, super greenhouse conditions^7^ and shallow shelf ocean anoxia^58^ suppressed initial recovery in the benthic realm resulting in very low beta diversity caused by turnover whereby widespread high abundance, low diversity communities of cosmopolitan ‘disaster taxa’, such as the foraminifera *Earlandia* and *Postcladella*^59^, bivalve *Claraia*, and brachiopod *Lingula*^39,60,61^, replace the incumbent Palaeozoic faunas. Conversely, nektonic diversity, amongst ammonoids, conodonts and fishes, seemingly recovered quickly in the Griesbachian (i.e. the first substage of the Induan stage). This recovery within the water column was short-lived with suggestions of a further extinction amongst nektonic groups occurring by the end of the Griesbachian^23,40^.

The subsequent Dienerian substage brought slightly cooler temperatures and less widespread anoxia and thus corresponded with some recovery which peaked simultaneously with lower oceanic temperatures at the Dienerian-Smithian (i.e. Induan-Olenekian) boundary^7,62^. This interval also coincides with a large positive carbon isotope excursion suggesting elevated levels of primary productivity^38^, possibly stimulating high diversification rates amongst planktotrophic nektonic groups such as ammonoids and conodonts^23^. Habitable area and resources in the marine environment increased with falling temperatures, increased oxygenation and nutrient fluxes^23,40^. However, other research suggests an “Induan-Olenekian boundary” or “Dienerian” crisis which supposedly corresponded with increased dysoxia^63^ and/or a negative carbon isotope excursion driven by a period of renewed volcanic activity, which preferentially affected benthic taxa^64^. However, quantitative evidence of this biotic event is currently lacking and the negative carbon isotope excursion appears to be regional, not global^65^.

The late Smithian witnessed a major environmental and biotic crisis which put an immediate stop to marine ecosystem recovery following the weak recovery in the Dienerian-early Smithian^66,67^. This Late Smithian Thermal Maximum event saw temperatures return to extreme greenhouse levels, with tropical sea surface temperatures (SSTs) reaching in excess of 38 °C^7,68^. In addition, a major negative carbon isotope excursion was observed ^38^ as well as an increase in ocean stratification and anoxia^29,65^. These perturbations drove heightened levels of extinction, particularly in nektonic groups such as conodonts and ammonoids^23,40^.

The Spathian substage heralded a sustained interval of ecosystem recovery and increased beta diversity which persisted through to the Middle Triassic, which is when some consider full marine ecosystem recovery to have been achieved (both globally and regionally)^69^. This interval saw SSTs reducing significantly (to 30–32°C in the tropics)^7^ and a decline in the extent of ocean anoxia. This led to rediversification of benthic clades, trace makers and pelagic organisms – some of which were new groups occupying high trophic levels (e.g. marine reptiles)^51,58,69^. The more sustained levels of recovery seen in the Spathian-Anisian relative to the earlier Triassic have been linked to the longer period of environmental stability once this eruptive phase of the Siberian Traps LIP had ceased^70^. It is widely considered that the final stage of the recovery of ecosystem complexity took place on the continental shelf by the mid-late Anisian, some 8–10 million years after the PTME^69,71^ with the recovery of metazoan reef systems^72^ and introduction of new predators occupying previously vacated higher trophic levels represented by a diverse marine reptile fauna that had no analogue in the Permian.

## Hypotheses for ecosystem recovery

The recovery of the marine biosphere after the PTME has always been considered to be prolonged^37^. Initial attempts to quantify recovery via the re-attainment of alpha diversity within communities and global generic richness (i.e. gamma diversity) place full recovery, at the earliest, in the Middle Triassic – some 5 million years post PTME^47,48^. However, taxonomic and ecological recovery can be decoupled, and full recovery of functioning marine communities may have taken longer, stretching the recovery interval from the PTME further into the Mesozoic^49,50,69^.

**Fig. 2:**
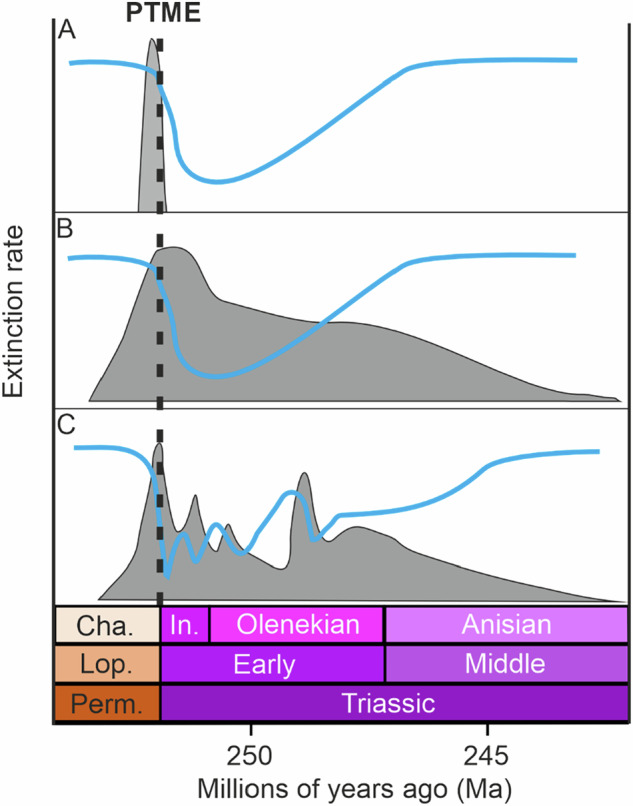
Schematic adapted from Wei et al. (2015)^29^ showing different hypotheses explaining the long recovery interval from the PTME. **A** Recovery (blue line) is suppressed by the intensity of the mass extinction (grey curve) which caused very low species diversity driving ecological undersaturation and stifled diversification due to lack of competition. **B** Recovery (blue line) is suppressed by prolonged harsh conditions (grey curve) throughout the Early Triassic. **C** Recovery (blue line) occurs rapidly under favourable conditions but is slowed or stopped by episodic environmental perturbations throughout the Early Triassic.

Chen and Benton^69^ hypothesised that ecological recovery occurred in a step-wise, bottom-up fashion from lower to higher trophic levels (Fig. 1D) with communities in the immediate aftermath of the mass extinction consisting of just the basal tiers of the trophic pyramid (i.e. primary producers and primary consumers – the classic disaster taxa assemblages). Ecosystem recovery then occurred with re-establishment of higher trophic levels (i.e. secondary and tertiary consumers) through the rest of the Early Triassic and Middle Triassic, re-building the trophic pyramid step-by-step, with the full recovery of communities happening by the mid-late Anisian, corresponding to the filling of apex predator niches (i.e. trophic level 5) by marine reptiles and large fishes^69,71^. This hypothesis relies on scenarios represented in Fig. 2A and B, whereby biotic recovery occurred slowly but in a stepwise manner due to delayed and then gradually ameliorating environmental conditions and ecosystem undersaturation brought about by the sheer magnitude of the mass extinction losses.

In contrast to the stepwise ecosystem rebuilding hypothesis of Chen and Benton^69^, there is sporadic evidence for complex, multi-trophic level communities in every substage of the Early Triassic^73,74^, albeit punctuated by periods of environmental perturbation that might have caused further ecosystem collapse or at least temporary slowing or cessation of ecological recovery^58^ (Fig. 1C). On one hand, such evidence casts doubt on scenarios of delayed diversification due to ecosystem undersaturation^34^ and prolonged abiotic stresses^37^. This suggests rapid recovery of communities and wider ecosystems during geologically short periods of favourable conditions between perturbation events^58^. On the other hand, these supposed highly complex communities appear to show low levels of alpha diversity, particularly in lower trophic levels. This suggests that, whilst certainly not being restricted to only primary producers and primary consumers, these early post-extinction ecosystems were not yet fully recovered as they show low levels of functional redundancy which could result in lower levels of interspecific competition thus aligning with the ecosystem undersaturation hypothesis of slow recovery rates^34^.

Observing marine ecological structure across broader temporal scales reveals further interesting patterns that suggest that the ecological recovery interval across all marine habitats might have extended into the Late Triassic^50^. Analyses of the balance between the diversity of benthic/pelagic and motile/non-motile taxa showed that the Early Triassic displays an inverted functional pyramid compared to the Late Permian and Middle–Late Triassic intervals, with highly diverse motile pelagic communities and depauperate benthic communities^50^ dominating the Early Triassic (Fig. 1B)^73,74^. This can be attributed to widespread harsh benthic conditions (i.e. anoxia) and the rapid boom-bust diversification of pelagic clades such as ammonoids and conodonts in the Early Triassic and shows that ecological recovery can manifest in different ways, at different spatial and temporal scales. A closer inspection of the more complex ecosystems of the earliest Triassic^73^ reveals that, although they are functionally diverse, they appear to have low levels of functional redundancy, particularly at lower trophic levels. This suggests that although communities were not rebuilt step-by-step throughout the Early–Middle Triassic, as suggested by Chen and Benton^69^, they may not have attained levels of advanced ecological recovery and were instead manned by so-called skeleton crews^47,52^. This can be seen throughout the Early Triassic and could be a result of repeated abiotic perturbations or the prolonged stress of anoxia and high temperatures on benthic communities^50,75^. This may have prevented the recovery of functional redundancy and thus ecosystem saturation and stability^34,50^. Whilst the Middle Triassic functional pyramid appears to be returning to similar levels to pre-extinction times (i.e. greater diversity of benthic taxa), it was not until the Late Triassic where the balance between different modes of life in the ocean returned to levels seen in the Permian^50^ (Fig. 2B), a pattern that is seemingly reproduced in the aftermath of the subsequent Late Triassic mass extinction in the earliest Jurassic^52^.

## Recovery or restructuring?

The PTME represented the biggest ecological upheaval in the oceans in Earth’s history^55^ and witnessed the switch of dominance from Palaeozoic to modern ocean faunas^45^. Therefore, it can be suggested that the rebuilding of marine ecosystems in the Triassic can be referred to as “restructured” rather than “recovered” because the tiered, benthic epifaunal communities of the Palaeozoic were replaced by communities of increasingly motile and infaunal animals^76^. It has been suggested that this change is referred to as a rebound rather than a recovery, whereby ecological regime shifts play out as ecosystems return to stable states but with new clades attaining ecological dominance whilst others are relegated to more marginal roles^43^. It has thus been hypothesised that the PTME was the main trigger for the origins of modern marine ecosystem structure^4^, whereby Meso-Cenozoic marine ecosystems exhibit greater complexity driven by changes in functional structure^77^. In fact, the PTME may have been the initial catalyst of the Mesozoic Marine Revolution (MMR)^25^, the diversification of predatory clades which caused an escalation event of predator-prey arms races through the Mesozoic and Cenozoic^78^. Restructuring rather than recovery is also supported by ecological metrics^49^ and clear ecological regime shifts such as the shift in dominance in benthic communities from brachiopods to molluscs^79^ and the Triassic origination of new groups such as marine reptiles^69^. All this evidence points to major structural differences between Palaeozoic and Mesozoic marine communities given that ecological structure is defined by the composition (i.e. origination of new Mesozoic taxa), and evenness (i.e. shift in balance between major clades from Palaeozoic to Mesozoic faunas) of organisms within a community and the interactions (i.e. escalation associated with the MMR) between them.

Different metrics for measuring rebound/restructuring hint at different rates of ecosystem rebuilding after the PTME and different models hypothesise different scenarios in how the reestablishment of ecosystem complexity took place^42,47,49,50,69^. Direct fossil evidence and environmental proxies from the Early–Middle Triassic cast doubt on extinction magnitude and prolonged environmental stress being solely responsible for suppressing global recovery rates. The occurrence of sporadic Lägerstatten provides evidence of complex communities of multiple trophic levels appearing in the fossil record just 1 million years after the PTME^73^. This evidence suggests the possibility that initial recovery of marine ecosystems happened quickly in the aftermath the PTME, at least in some parts of the world^73,74^. However, these post-extinction communities appear dominated by pelagic animals^50^, and this interval of early recovery was likely delayed, suppressed, and possibly stopped by repeated environmental perturbations throughout the Early Triassic^40,58,73,80^. Later recovery in the Middle Triassic hints at restructured Mesozoic marine ecosystems^49,76^ and heralds the onset of the MMR and possibly the origins of modern marine ecosystem structure^81^.

To further our understanding of how the biosphere bounced back from the PTME in the ocean, we need a more comprehensive sample of community-level data sets from the fossil record, spanning different latitudes and ocean basins. In addition, methods used for quantifying ecosystem recovery/restructuring from the PTME have been inadequate for capturing community-level processes (e.g. biotic interactions/population sizes) that influence ecosystem structure, function, and stability. Sophisticated approaches^13,57,82,83^ that encompass biotic interactions and how they mediate community collapse and recovery will prove pivotal in our understanding of extinction and recovery dynamics in the distant past and how they can be used to help us predict biotic response to disturbance in our present day and future oceans.

## Data Availability

No datasets were generated or analysed during the current study.
